# Applied Artificial Intelligence in Healthcare: A Review of Computer Vision Technology Application in Hospital Settings

**DOI:** 10.3390/jimaging10040081

**Published:** 2024-03-28

**Authors:** Heidi Lindroth, Keivan Nalaie, Roshini Raghu, Ivan N. Ayala, Charles Busch, Anirban Bhattacharyya, Pablo Moreno Franco, Daniel A. Diedrich, Brian W. Pickering, Vitaly Herasevich

**Affiliations:** 1 Division of Nursing Research, Department of Nursing, Mayo Clinic, Rochester, MN 55905, USA; nalaie.keivan@mayo.edu (K.N.); raghu.roshini@mayo.edu (R.R.); ayala.ivan@mayo.edu (I.N.A.); charlieobusch@gmail.com (C.B.); 2 Center for Aging Research, Regenstrief Institute, School of Medicine, Indiana University, Indianapolis, IN 46202, USA; 3 Center for Health Innovation and Implementation Science, School of Medicine, Indiana University, Indianapolis, IN 46202, USA; 4 Department of Anesthesiology and Perioperative Medicine, Mayo Clinic, Rochester, MN 55905, USA; diedrich.daniel@mayo.edu (D.A.D.); pickering.brian@mayo.edu (B.W.P.); vitaly@mayo.edu (V.H.); 5 College of Engineering, University of Wisconsin-Madison, Madison, WI 53705, USA; 6 Department of Critical Care Medicine, Mayo Clinic, Jacksonville, FL 32224, USA; bhattacharyya.anirban@mayo.edu; 7 Department of Transplantation Medicine, Mayo Clinic, Jacksonville, FL 32224, USA

**Keywords:** healthcare, hospital, computer vision, artificial intelligence, system

## Abstract

Computer vision (CV), a type of artificial intelligence (AI) that uses digital videos or a sequence of images to recognize content, has been used extensively across industries in recent years. However, in the healthcare industry, its applications are limited by factors like privacy, safety, and ethical concerns. Despite this, CV has the potential to improve patient monitoring, and system efficiencies, while reducing workload. In contrast to previous reviews, we focus on the end-user applications of CV. First, we briefly review and categorize CV applications in other industries (job enhancement, surveillance and monitoring, automation, and augmented reality). We then review the developments of CV in the hospital setting, outpatient, and community settings. The recent advances in monitoring delirium, pain and sedation, patient deterioration, mechanical ventilation, mobility, patient safety, surgical applications, quantification of workload in the hospital, and monitoring for patient events outside the hospital are highlighted. To identify opportunities for future applications, we also completed journey mapping at different system levels. Lastly, we discuss the privacy, safety, and ethical considerations associated with CV and outline processes in algorithm development and testing that limit CV expansion in healthcare. This comprehensive review highlights CV applications and ideas for its expanded use in healthcare.

## 1. Introduction

The use of technology to address inefficiencies within the healthcare system and optimize patient safety has an extensive history of development, starting with the documentation and recording of patient care events. The concept of the electronic health record (EHR) emerged in the 1970s with the first official EHR built in 1972 by the Regenstrief Institute at Indiana University and has grown expediently over several decades [1,2]. The EHR provides a historical record of the patient care that was ordered and completed, making the EHR inherently retrospective. While this historical record is necessary for legal, administrative (billing), and diagnostic confirmation (post-test probability), it is cumbersome to use for real-time clinical decision-making like predictions, detection, and prognosis. This limitation leads to the inability to anticipate the healthcare needs of the patient, as well as the disease process that may be occurring; this is otherwise known as pre-test probabilities [3]. An opportunity to obtain more granular, real-time data is to use ambient sensors such as CV. CV mimics human vision that integrates and interprets visual information and could be potentially used to create sophisticated algorithms in real-time.

We recognize that much can be said about CV in terms of development trends and internal causal relationships of the overall and individual application of this technology. However, the purpose of this manuscript is to provide a high-level, comprehensive overview of the application of CV in healthcare settings based on other industries. First, we will briefly review the use of CV in industries outside of healthcare and categorize its application into themes. Following these themes, we review in greater detail how CV has been applied and/or developed for the hospital setting, and then we review outpatient and community settings. To identify future opportunities for the application of CV, we completed journey mapping at the patient, clinician, and system levels. Lastly, we discuss the privacy and safety considerations and ethical implications for the use of CV in the healthcare setting.

## 2. Overview of Computer Vision

CV is a type of artificial intelligence (AI) that uses digital videos or sequences of images. The goal of CV is to train computers to extract information from the images, essentially enabling computers to “see” and recognize content [4]. The foundations of CV were established during the 1980s; they were marked by the development of algorithms like optical flow and edge detection [5,6]. The advancement of machine learning and statistical techniques in the 1990s empowered computer applications to acquire the ability to understand and process more intricate patterns within visual scenes [7,8]. During the 2000s, the application of CV manifested in more practical domains, including the analysis of medical images and the detection of faces. The use of Convolutional Neural Networks (CNNs) significantly advanced the field of CV in 2012 when CNNS demonstrated high performance in the ImageNet Large Scale Visual Recognition Competition [9], emerging as the predominant learning method in CV. CNNs showed expert-level performance in image classification, object detection, and semantic segmentation across diverse fields, including medicine, surveillance, and autonomous driving [10,11,12,13,14,15,16]. As machine learning models advanced, obtaining a sufficient quantity of labeled data became a challenge. In response to this obstacle, unsupervised techniques like clustering and dimensionality reduction were developed. These approaches delve into the inherent structure of data without relying on explicit guidance to address the scarcity of labeled data [17,18,19]. However, the adoption and therefore application of CV into hospital settings has been slow. Its utilization in healthcare comes with limitations, as ethical and privacy concerns take precedence when involving humans, particularly humans in a vulnerable state (i.e., patients). As CV continues to develop, including the potential to assist in many aspects of patient care (e.g., documentation, recognition of a deteriorating patient, etc.), it is important to revisit how CV could be utilized for the benefit of patients and providers.

CV encompasses a multidisciplinary domain integrating advanced machine learning techniques, pattern recognition, and image processing to empower computers to comprehend the visual content present in images and videos [16,20,21,22]. Typically, CV algorithms start with the acquisition of data through cameras or sensors, followed by preprocessing and image enhancements. CNNs play a pivotal role in automatically learning the representations of visual scene content and contribute significantly to various CV tasks. Due to the CNNs’ robust feature representation capabilities, CNNs have found widespread application as an effective method for extracting meaningful patterns and features [23,24,25]. Despite the complexity of training CNNs due to their numerous layers, the artificial intelligence field addresses these challenges by adopting transfer learning and fine-tuning techniques to enhance model efficiency and representation power [26,27,28]. Object detection, crucial in applications like medical diagnostics and autonomous driving, heavily relies on CNNs, where a CNN backbone network extracts image features, and candidate regions determine target category and location information. CNNs also find applications in object classification across medical imaging, security, and agriculture, among other industries. For instance, in medical imaging, CNNs extract relevant features for categorizing structures such as tumors, aiding in diagnostic processes [29,30,31]. By means of parallel processing, Graphics Processing Units (GPUs) (i.e., computer chips) enhance the efficiency of managing extensive matrix operations essential for the processing of CNNs. This parallel approach substantially diminishes training times, thereby streamlining the implementation of real-time applications utilizing CNNs [32]. The evolution and implementation of CV have undergone notable transformations in the utilization of deep learning models and the expansion of machine learning methods. These deep learning models and methods are outlined further in Table 1.

## 3. Application of Computer Vision in Industry Outside of Healthcare

The use of CV was identified in 24 major industries including agriculture, engineering and manufacturing, retail, and education, among many others. The application of CV largely fell into four different themes or categories: job enhancement, surveillance and monitoring, automation, and augmented reality. These themes are outlined in Table 2 and provide insight into how CV could be applied in healthcare. Examples of job enhancement include the use of CV to analyze sporting events to inform referee calls [33], the scoring of diving competitions [34], and insurance appraisals to assist with claim reporting [10]. The use of CV in surveillance included the detection of forgery in artwork [35,36,37] as well as other industries, the prevention of cheating in academic and educational settings, and the enforcement of speed limits [14]. The theme of monitoring was the largest, including the use of CV to monitor agricultural crops for disease or insect infestations [15,38,39], detect restocking needs in warehouses or retail stores [40], and identify defective products on assembly lines [41]. The final category of augmented reality included technology such as Apple Vision [42], the ability to try clothes on virtually [43,44,45], and tools to assist people with vision impairment and blindness [46,47,48].

## 4. Computer Vision Application in Hospital Settings

Current medical applications of CV largely focused on monitoring (detection and measurement) and were mostly in the development and testing phases. The application or use of CV in the hospital included several commercial companies that specialized in patient monitoring for falls (Artisight: https://artisight.com/, accessed on 18 January 2024, CareAI: https://www.care.ai/sensors.html, accessed on 18 January 2024, Inspiren: https://inspiren.com/solutions/, accessed on 18 January 2024, Ocuvera: https://ocuvera.com, accessed on 18 January 2024, VirtuSense: https://www.virtusense.ai/, accessed on 18 January 2024) [88,89,90,91,92], Magnetic Resonance Imaging (MRI) and Computed Tomography (CT) support (Philips: https://www.philips.com/a-w/about/artificial-intelligence/ai-enabled-solutions, accessed on 18 January 2024; Silo AI: Europe’s largest private AI lab|Silo AI, accessed on 18 January 2024) [93,94], and patient and protocol monitoring including hand sanitation (CareAI). CareAI also advertises automated natural language processing [88]. Published peer-reviewed literature on the effectiveness of these models or documentation of the implementation process into clinical care is scarce. Ocuvera includes an overview of pre- to post-implementation fall data with significant differences in fall rates [90]. Inspiren and Virtusense provide case studies and white papers that overview the technology [91,92]. Philips outlines the science behind the algorithms in a series of research articles [93]. Outside of these companies, we identified several studies reporting on the development and application of CV to assist with radiology exams (i.e., X-rays, MRIs, CTs, and PET scans) for abnormalities signaling a disease process such as breast cancer [95,96,97]. The use of CV in radiology and histology is discussed next as these tools are either applied in practice or are closer to application. Table 3 emphasizes key domains of CV advancement in healthcare, detailing the types of images and deep learning models used.

### 4.1. Radiology

The use of CV in radiology has gained increased attention as it can support timely intervention and enhanced efficiencies within clinical workflows. For example, a recent study demonstrated how CV could support surgeons in diagnosing wrist fractures in pediatric patients [154]. The motivation for this type of CV application is to expedite surgical care in low-resource environments where specialized radiologists are not readily available. Another example of a clinical application is notification software (AIDOC), which is used to detect intracranial hemorrhage [141]. Teleradiology networks can employ this software to expedite stroke workups in critical access hospitals or lower trauma centers. Other CV radiology applications concern detecting anomalies within medical images. A recent study by Lakhani et al. (2017) [99] employed deep models such as 2D-CNN, AlexNet [9], and GoogleNet [227] in their CV approach to detecting tuberculosis in chest radiographs and pulmonary tuberculosis in chest radiographs. Other studies applied ensemble learning for the diagnosis of Alzheimer’s Disease using MRI brain images [139,140,142]. The typical image types utilized for radiology segmentation include X-rays, CT scans (i.e., liver tumor, [138]), MRIs (i.e., brain tumors [149]), and 4D-CT (i.e., brain tissue for stroke workup [148]). While the utilization of deep learning has resulted in precise detection rates in the field of radiology, these approaches require extensive, well-annotated datasets. Without such datasets, deep learning methods may experience overfitting, leading to a reduction in their generalizability [228]. There are a multitude of other examples of CV applications in radiology as shown by recent reviews including emergency radiography, stroke workup, and workflow efficiencies [228,229,230]. The implementation and use of these algorithms have been slower than expected and may be due to a lack of standard user interfaces and differing expectations among clinicians and administrators as reported in a recent study [231].

### 4.2. Histology

The examination of histological images by pathologists provides diagnostic information crucial for influencing a patient’s clinical outcome. Traditional histological image representation involved extracting texture and color features and employing conventional machine-learning approaches. However, the CV landscape has evolved with the extensive representational power offered by Convolutional Neural Networks (CNNs) [127,134]. For example, Bejonrdi et al. (2017) [112] demonstrated that the application of CNNs for detecting lymph node metastases in breast cancer outperformed eleven pathologists in a simulated time-constrained setting. Tellez et al. (2018) [113] developed a CNN-based CV approach capable of effectively detecting mitosis in Hematoxylin and Eosin whole-slide images . Additionally, Kather et al. (2019) [136] showcased that microsatellite instable tumors in gastrointestinal cancer could be directly predicted from H&E histology using CNNs to classify tumors versus normal tissues. The present challenge involves handling high-resolution histological images, requiring substantial computational resources and extensive training sets. Employing transfer learning and knowledge distillation approaches may partially mitigate this challenge [232].

## 5. Development and Testing of Computer Vision in the Hospital Setting

Several CV-based tools were identified that had been shown to be effective in the testing and development phases; however, they were not yet put into practice at scale. These include the detection of behaviors and signs related to delirium, pain detection and monitoring, monitoring of sedation depth and signs of patient deterioration, mechanical ventilation, and monitoring of the care setting aimed at improving patient safety and quantifying workload. We provide an overview of these studies in this section.

### 5.1. Detection and Monitoring of Brain Health

Delirium, a type of acute brain dysfunction, occurs in 50–80% of critically ill patients [233]. An observational pilot study reported that delirious patients had significantly different expressions of facial action units, head pose movement, and other environmental factors such as visitation frequency measured by CV (*n* = 22) [156]. A different study examining the frequency of caregiver actions reported that delirious patients had more caregiver activity overall, which was most concentrated from 8:00 p.m. to 12:00 a.m. [195]. These observational differences between non-delirious and delirious patients can be automated to aid in the recognition of early warning signs of delirium, the measurement of delirium severity, and aid in subtyping and phenotyping efforts.

### 5.2. Detection and Quantification of Pain, Agitation, and Level of Sedation

CV has been used to detect pain in a variety of patient populations (infants to aging adults), specific disease states (lung cancer, dementia, chronic back pain, shoulder pain), and after procedures (procedural pain in infants), mostly in community or outpatient settings. A recent scoping review identified one study that tested the feasibility of an automated approach to pain assessments using a deep-learning method in a population of critically ill patients [176,177]. The study tested the accuracy of models to dichotomize pain and rate pain on three levels (0–2), reporting >0.85 accuracy for dichotomized models compared to 0.56 for the three-level model [176].

Automated pain recognition and monitoring have been widely explored in neonatal populations. Several reviews report on CV developed over the past two decades aimed at the recognition of pain, monitoring, and the measurement of intensity [159,163,171,179]. These feasibility studies and developed models have cumulated into a point-of-care mobile health application for procedural pain named PainChek Infant [172]. In a feasibility study of forty infants (age range 2.2–6.9 months), the mobile health application significantly correlated (r = 0.82–0.88, *p* < 0.0001) with clinician-administered pain scales (Neonatal Facial Coding System and Observer administered Visual Analogue Scale) and demonstrated high interrater reliability (ICC = 0.81–0.97, *p* < 0.001) and high internal consistency (alpha = 0.82–0.97) [172]. This type of technology could be applied to adult hospitalized patients to improve pain monitoring and assist clinicians with follow-up pain assessments after the administration of analgesics, especially in patient populations that are not able to verbalize their discomfort and pain level. Furthermore, this type of state-of-the-art technology was emphasized by a recent narrative review that discussed the updated definition of pain from the International Association for the Study of Pain and how multidimensional technologies are needed to improve the identification and monitoring of pain [234]. Untreated pain can result in delirium, agitation, hostility, and other adverse consequences such as impaired healing and increased mortality risk [235,236,237]. The use of CV, paired with other artificial intelligence modalities, clinicians, and patients within a model, could improve the proactive recognition and monitoring of pain in hospital environments across populations.

Proof of concept CV models have been developed to recognize and monitor facial and body movements associated with agitation during sedation, such as grimacing. One study used a volunteer to simulate agitation through different levels of grimacing, developing a proof-of-concept algorithm that can be tested in critically ill patients [158]. Another study used a volunteer to simulate limb movement during agitated episodes and then tested this model on five ICU patients. The results of the model were correlated over time with a nurse-administered Riker Sedation Agitation Scale and physiologic signs including heart rate, heart rate variability, systolic blood pressure, and blood pressure variability [157]. Lastly, a study on young children and infants used eye movements to facilitate the measurement of sedation and consciousness levels in young children and infants [168].

### 5.3. Patient Deterioration

The facial action unit is a comprehensive system that conveys facial movements, subsequently utilized for detecting emotions such as anxiety, stress, fear, or pain [164,166,173]. As presented in reference [175], Giannakakis et al. (2022) illustrated that facial action units are associated with stress levels. This implies that during stressful situations, specific action units, such as cheek raising, lid tightening, and upper lip raising, intensify.

The early warning signs of an impending acute patient deterioration are often subtle and overlooked by busy clinical staff leading to a delay in the escalation of care [238]. To address this limitation, a recent feasibility study examined how subtle changes in facial expressions were associated with a future admission to the intensive care unit. This study used CV to identify facial action units and reported that combinations of the upper face, head position, eye position, lips and jaw, and the lower face were associated with the increased likelihood of admission to the intensive care unit (*n* = 34 patients) [181]. This algorithm could be used to proactively identify patients at risk of acute deterioration and support early intervention [181]. In a post hoc analysis of these data, a decrease in the number of facial expressions (per time unit) and an increase in the diversity of facial expressions predicted admission to the ICU (AUC = 0.81) [182]. Numerous opportunities exist to expand on this CV algorithm and investigate how other signs, such as the frequency of clinician visits to the patient’s room or the presence of certain respiratory devices [183], could improve early recognition of impending acute deterioration.

### 5.4. Mechanical Ventilation

CV has been applied to the field of mechanical ventilation to estimate regional lung volumes using light to reconstruct the motion of the lungs and measure the regional pressure distribution [184]. This proof-of-concept model was developed by Zhou et al. using a mannequin that measured and monitored chest expansion with a light projector and cameras. They utilized surface reconstruction of regional chest expansion for their model, which showed good accuracy with an error of 8 mL under 600 mL tidal volume. They compared their methods with other frequently used computational models and reported a 40% reduction in computational costs paired with improved accuracy in their new model. This work needs to be clinically tested and validated.

### 5.5. Mobility

The use of CV to monitor and document physical activity and early mobilization in critically ill patients was reported in two studies. CV was used in a recent study to develop a tool for real-time patient mobility monitoring in the ICU [187]. Yeung et al. (2019) reported a mean sensitivity of 87.2% and specificity of 89.2% with an AUROC of 0.938 for detecting mobility activities (i.e., get out of bed, get into bed, get out of chair, get into chair). The CV had an accuracy of 68.8% for quantifying the number of healthcare personnel involved in each activity [187]. Another study by Reiter et al. (2016) developed an automated mobility sensor to support the monitoring of patient activity in the surgical ICU. They compared the algorithm’s performance with clinician performance on the identification of physical activity and reported high inter-rater reliability with a weighted Kappa score of 0.86 [186]. These types of models could automate documentation of physical activity in hospital patients, decreasing clinician documentation burden and increasing the accuracy of electronic health records.

### 5.6. Patient Safety

Several studies have developed models focused on improving patient safety using CV as a monitoring tool. One focus is hand hygiene, an essential component of infection prevention. While compliance is critical for patient safety, monitoring clinician performance is time-consuming. It can be inaccurate as it requires human observation of care procedures both inside and outside the patient room. CV provides an opportunity to automate monitoring. This use case has been demonstrated using depth sensors in a hospital unit and video and depth images in a simulated hospital room [192,220]. Both models achieved sensitivity and specificity greater than 90% in detecting hand hygiene dispenser use and performed better than human observers. In addition to monitoring, future studies could explore how the model could provide real-time feedback to clinicians, or reminders of hand hygiene, leading to further opportunities to improve patient safety.

Surgical procedures and operating rooms (ORs) are the focus of a recent review that highlights how CV could improve patient safety and system efficiencies [198]. A recent study used off-the-shelf camera images to measure the level of situational awareness of surgical teams during timeout procedures in the OR. The model distinguished between teams with good and poor situational awareness, substantiating existing studies in the OR on the use of CV to augment traditional human-based observation assessments [194,198]. Other CV-based models have aided in surgical phase recognition, robot-assisted surgeries, surgical skill assessment, detection of instruments or lesions during surgery, enhanced visual displays in surgeries, and navigation during surgical procedures [194,198,199].

### 5.7. Quantification of Workload in the ICU

A few studies have demonstrated the feasibility of ambient monitoring of caregiving activities in the ICU using CV. The first study completed a task recognition of caregiving activities over 5.5 h with an accuracy of 70% in a pediatric ICU [226]. The recognized tasks included documentation, observation, and procedures, among others [226]. These tasks were then examined over time for trends. The second study recognized and then categorized patient and caregiver movement (i.e., workload) over the course of 24 h in an adult ICU [195]. The study reported significant differences in patient and caregiver movement throughout the 24 h period, between intubated and non-intubated, delirious and non-delirious, and settings (high dependency unit vs. ICU). Another study developed and validated a Clinical Activity Tracking System (CATS), testing its use in both a simulated and actual ICU room. Like the previous study, more caregiving activity was reported between 7:00 a.m. to 11:00 p.m. compared to 11:00 p.m. to 7:00 a.m. [221]. This system was validated against manual observation with a correlation of r = 0.882 [222]. Improving the quantification and understanding of caregiver workload and function throughout a time period was the focus of these studies as existing monitoring systems are resource intensive and subjective [195,222,226].

## 6. Computer Vision in Outpatient and Community Settings

CV has been developed in the community and outpatient settings to detect, measure, and monitor patient symptoms, signs of underlying illness or disease, and patient events such as falls. A recent survey identified over thirty different CV models developed to automatically detect underlying symptoms related to medical diagnoses [162]. These include monitoring vascular pulse, pain, facial paralysis, neurologic conditions, neurodevelopmental disorders, psychiatric disorders (i.e., attention deficit hyperactivity disorder (ADHD), autism, depression), and mandibular disorders, among others [162]. This type of computer-assisted diagnosis ranges from the detection of facial shape, facial features, and facial muscular response to voluntary emotion and facial motion analysis. In this section, we briefly overview the subject areas with the highest level of development.

### 6.1. Pain Detection and Monitoring in Community Settings

The automatic recognition and monitoring of pain in the community and outpatient setting is well-established. A systematic review in 2021 (*n* = 76 studies) reported on the use of CV to diagnose and treat chronic lower back pain [167]. Also, in 2021, a survey of automated detection of pain summarized studies and CV across populations, providing an in-depth overview of datasets, learning approaches, spatial representations, and machine learning methods used [170]. A narrative review highlighted the state-of-the-art technology published on pain detection and monitoring [234]. Lastly, a scoping review reported on several community and outpatient models to detect and monitor pain using CV [177]. A recent study used recent developments in CV automated segmentation and deep learning along with the updated definition of pain from the International Association for the Study of Pain to develop a sentiment analysis system within a Smart Healthcare System for pain monitoring [180]. This CV model and most models mentioned in the reviews need to be prospectively tested [170].

### 6.2. Neurologic, Neurodevelopmental, and Psychiatric Disorders

Autism spectrum disorder, a neurodevelopmental disorder, is increasingly prevalent in pediatric populations [239,240]. Time to diagnose and receipt of needed care and resources can be delayed by months, leading to deficiencies in care. To address this gap in clinical care, an interactive, mobile health technology was developed through a series of studies that uses CV in a closed-loop system to automatically code signs and behaviors associated with autism [202]. The intent is for parents to use this technology at home to improve the early recognition of autism and access to needed resources and care. This use case and framework could be expanded to include additional neurodevelopmental disorders with similar impact. A different study developed a CV model that could differentiate between individuals with autism spectrum disorders, ADHD, and healthy controls [200]. Head motion and facial expression were used to distinguish between these disorders [200].

The detection and severity of depression have been automated using CV in a few studies [174,178]. Depression is one of the most common psychiatric disorders and is often underrecognized, leading to delays in patient care and decreased quality of life. The application of CV to detect and measure depression could have widespread implications and lead to the early detection and allocation of resources to improve patient care. For example, such an algorithm could be applied in telehealth outpatient visits where depression may not otherwise be discussed or during a hospitalization where situational depression can increase patient stress and lead to prolonged hospitalization and readmissions. In addition to depression, one study explored the accuracy between clinician-rated and computerized recognition of blunted facial affect [209,241].

To detect facial weakness, Zhuang et al. (2020) developed CV using images and videos of people collected from Google Images and YouTube videos [203]. Six neurologists used a rating scale (likelihood of facial weakness) to label the images. Following model development, the authors concluded that the combination of landmark and intensity features led to the highest accuracy. The ability to detect the shape (i.e., landmark) and texture (i.e., gradient intensity) was contributed by the neurologists who labeled the images [203]. Facial palsy detection has been the focus of several studies. Guarin et al. (2018) retrained a CV facial landmark detection model that was previously trained using healthy individuals with facial palsy patients to develop a more accurate model [201]. Ruiter et al. (2023) studied the use of facial recognition software to identify patterns of facial weakness, and a deep learning model was trained for classification and disease severity in a cohort of myasthenia gravis patients [204]. The images used for training were collected in the outpatient setting. The area under the curve for diagnosis of myasthenia gravis was 0.82 and 0.88 for disease severity [204]. Another recent study assessed the intensity of facial weakness in patients with facial palsy. The intensity was classified into three levels by focusing on specific facial landmarks. The accuracy of detecting palsy patients was 95.61%. The accuracy for class assignment (intensity level) was 95.58% [205].

To improve virtual interactions and patient education efforts, a CV algorithm detected changes in facial expressions indicative of confusion and compared its accuracy to forty medical students [189]. The accuracy of the human raters in identifying confusion was 41% compared to 72% accuracy by the CV algorithm using four different facial action units (lowered brow, raised cheek, stretched lips, and lip corner pulled).

### 6.3. Falls

With the increasing population of aging adults and their preferences to live at home, falls at home have contributed to a significant increase in the risk of morbidity and mortality in the population [242]. To address the increasing incidence of falls, CV technology has been developed to detect and monitor for risks and signs of falls in the home environment. A literature review completed in 2023 surveyed the use of ambient sensors to detect falls in the home environment. While some studies have used CV to detect falls, other systems are a combination, or hybrid, of wearable and ambient sensor technologies [216]. One example of CV fall detection was developed by Joshi et al. (2017) [215]. A single camera was used to detect four different movements indicative of a fall event, and notifications were sent via email to the designated individual if a fall was detected. The CV model achieved an accuracy of 91.8% [215]. This model, and the majority identified by the recent review, focused on the detection of falls and not on the prediction or identification of early warning signs [216].

## 7. Journey Mapping and Future Computer Vision Application

**Figure 1:**
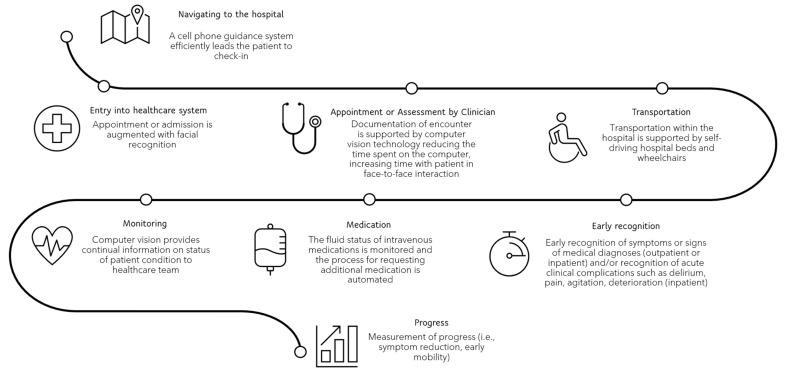
The completed journey mapping of the patient and clinician through the healthcare system. This figure depicts a scenario demonstrating the application of computer vision in a hospital setting. Each data point addresses a specific instance where computer vision could effectively enhance the system, optimizing the patient’s experience and the clinical workflow. This system would aim at directing time and resources more efficiently towards patient care management and improvement of patient outcomes.

**Figure 2:**
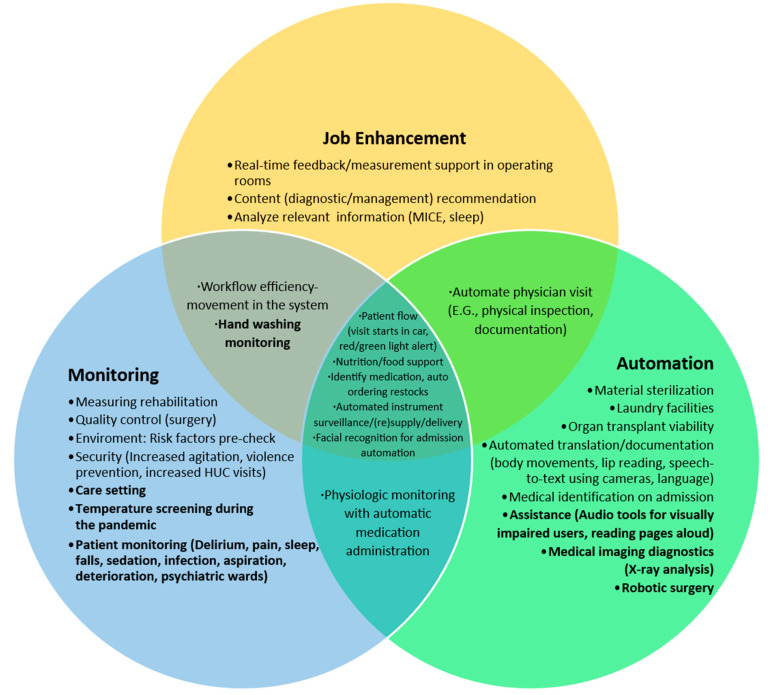
The Venn diagram displays how the main applications of computer vision in healthcare are organized. There are three central domains that we identified through our review: monitoring, job enhancement, and automation. In bold, attention is drawn to applications that are frequently mentioned in the literature. Overlap between categories is shown. The non-bold text highlights potential uses of computer vision that are either in development/testing stages or need proof-of-concept work to be completed.

To investigate how CV could be applied in the hospital setting, the temporal journeys of the clinician and patient through the healthcare system were mapped and analyzed for opportunities. The overview of the journey map is illustrated in Figure 1. Many identified opportunities to incorporate CV into patient care and the healthcare system overlapped. These identified opportunities are displayed in Figure 2. For example, the use of facial recognition technology to automate patient check-ins in the outpatient and inpatient settings improves the efficiency of the system while also providing a smoother process for the patient. The monitoring of parking lots for available patient parking and the use of interactive displays to provide directions to clinic or hospital appointments benefit the system and the patient. Individuals who were responsible for patient check-in could instead meet, greet, and accompany a patient on their clinic or hospital journey to improve the coordination of care. The integration of CV into clinic and hospital rooms could improve the monitoring of patient conditions, resulting in early detection of acute deterioration or patient discomfort, assist with diagnostic testing, provide real-time feedback on the effectiveness of interventions to ameliorate patient discomfort, and complete auto-documentation of patient care procedures. These examples benefit the clinician, patient, and efficiency of the system. When taking ideas such as these from concept to development, it is crucial to identify who the end-user is, who benefits from the model or service (which may be different than the end-user), what efficiencies are improved, and what unintended consequences may result once the algorithm is in production. Additionally, the privacy, safety, and ethical principles and values must be considered. These are discussed next.

## 8. Summary and Implications for Computer Vision Use in Healthcare

This rapid review identified a few applications of CV in the hospital setting. Most of the CV in hospitals is still in the feasibility and proof-of-concept stage, lagging behind other healthcare settings and industries. This gap in CV application in hospital settings is likely due to the availability of public datasets to train and develop models, data privacy and security needs, ethical considerations, and barriers inherent within a complex system.. We will discuss these limitations, including ethical and economic considerations. Our journey mapping exercise identified many future opportunities for CV in the hospital and outpatient settings. As future opportunities are considered, it is critical to understand what problem the CV aims to solve, the stakeholders involved in using it, how privacy, safety, and ethical concerns are addressed, and the potential unintended consequences of its use in these settings.

## 9. Data Privacy and Safety Considerations

Before using a CV model in the hospital setting, it is crucial to consider the data privacy and patient safety requirements. Privacy has multiple meanings that depend on the stakeholder’s perspective [243]. A recently published meta-synthesis highlights perspectives patients and health professionals share on the benefits and risks of artificial intelligence (AI) in healthcare [244]. A theme identified by patients and clinicians involved the importance of data security and use. Both stakeholder groups shared how the storage and protection of these data were essential to prevent records from being hacked and/or leaked. Further, the meta-syntheses reported that the unwarranted use of these data for commercial purposes was a significant concern [244]. These concerns are related to overall AI use in healthcare and are not specific to CV.

Privacy and data management concerns unique to CV center on the nature of ambient intelligence, how it is applied, and what information is captured in the video images [245]. As a recent perspectives article highlighted, it is important to collect the minimum amount of information needed to train and use the model [246]. This could mean using black and white images instead of color or blurring or removing unnecessary pieces that do not contribute needed information. It is also important to consider the inclusion of individuals who are not the focus of the model. For example, the patient may be the focus of data collection, but clinicians and visitors in the hospital room may also be included in the video capture. Everyone and everything within the image field is included in the data collection. It is important that all individuals who may enter the room are either consented (i.e., patient) or informed (i.e., clinician) of the data collection and what privacy protections are in place [246]. If the collected data are being considered for other purposes and the bystanders are reidentified, there should be a process in place to notify and gain consent of those individuals. These concerns emphasize the importance of data management (storage and use) within research studies, production teams, and the healthcare system.

Informed consent or assent of data collection detailing the why, how, and when behind the collection and the privacy protections in place is imperative to complete, particularly when capturing images of patients in a very vulnerable state. Decision points can be built into the consent process, allowing the patient to opt in to use their image data for other purposes. For example, an opt-in for sharing their images with external institutions or scientists can help facilitate the development of public image datasets that may accelerate CV development. It is important to clarify that once a model is in production, images do not need to be retained as it can operate as a closed-loop system. This may limit the transparency, or the ability to review the algorithm to understand the output, but it does improve privacy protections.

Safety considerations for CV in hospitals are multi-factorial. It is essential to consider the end-user of the model. How will the end-user use the information provided by the model in their decision-making? Who is responsible for maintaining the model to ensure its accuracy? CV models can improve patient and clinician safety. For example, a model could recognize the early warning signs of workplace violence and notify clinicians to improve their situational awareness and implement mitigation measures to prevent verbal or physical abuse. Another example that proof-of-concept models have demonstrated in the operating room setting is the detection of missed care, poor situational awareness, or procedure errors. Both examples would improve patient and clinician safety [194,198]. On the other hand, CV models could decrease the safety of patient care. Previous studies have shown how bias is readily introduced into models if the training data are not representative of a diverse population [247]. These biases can lead to embedded stereotypes, discrimination, and exclusion of certain patients [248]. Current deep learning models employed in CV tasks directly derive their knowledge from the training data. Consequently, the performance of the model is heavily influenced by the distribution of the training data. If bias is present in the training set, the model identifies it as a significant context, impacting the generative capabilities of the model for unseen examples. Numerous studies in the literature have explored methods to extract bias-independent feature embeddings, resulting in enhanced performance of neural networks when trained on biased datasets [249,250,251,252]. These methods can be integrated into model development along with representative sampling to minimize the risk of bias.

### 9.1. Ethical Considerations in Computer Vision

The use of CV in healthcare has broad ethical considerations that need to be addressed as algorithms and models are designed, developed, tested, and deployed. Each stage of the algorithm, including maintenance, should be considered, and continually re-evaluated to ensure the medical ethics of autonomy, beneficence, non-maleficence, and justice are upheld for the end-user. It is also important to define and consider who is the end-user (i.e., patient, a decision-maker for the patient, clinicians, administration, support staff) and proactively address ethical concerns [253]. Depending on the end-user and circumstances concerning the use of the technology, different ethical principles or values may need to be considered. A recent scoping review identified eleven different ethical principles and values on the use of artificial intelligence [254]. These include transparency, justice and fairness, non-maleficence, responsibility, privacy, beneficence, freedom and autonomy, trust, sustainability, and solidarity [254]. Similar themes along with societal implications were summarized in a recent narrative review by Elendu et al. (2023). Inherent within these principles is the importance of placing the patient at the forefront and ensuring that every patient has a fair and equitable opportunity to benefit from the technology [255]. This priority encapsulates the responsibility to ensure the model was built on a representative dataset that can be generalized broadly, i.e., any risk of bias, discrimination, and stereotyping is minimized, and the welfare of the patient is prioritized. To accomplish these goals, it is important to partner with a medical ethicist, sociologist, or patient-community stakeholder group to evaluate the technology from multiple viewpoints within an ethical framework [255]. Questions evaluating the intent of the model, who will use the model and who will benefit from the model, how the model will be implemented and maintained, the acceptability and usability of the model, the transparency of the algorithm and resulting decisions, who holds the ultimate responsibility for performance, and how unintended consequences will be identified, tracked, and evaluated are just a few topics that are essential to work through prior to the inception of the project.

### 9.2. Economic Considerations

Job replacement and loss are significant concerns regarding the application of artificial intelligence, including CV [256]. To ensure safety and ethical considerations are followed, it is important to build “human in the loop” models that use CV as a tool to inform decisions; however, the human is the critical decision maker on how to use the information provided [257]. CV should enhance processes to improve decision-making, efficiencies within the system, and patient outcomes. It should not replace humans. A framework for evaluating the implications of automation in artificial intelligence was shared in a recent working paper by the National Bureau of Economic Research [256]. This paper discusses the balance between potential displacement and increased demand in non-automated labor tasks that could enhance the human experience. This type of framework is important for healthcare systems to use as the adoption of CV is considered. A recent review studied how artificial intelligence models could result in healthcare cost savings over several years [258]. Although they reported significant cost savings with the use of artificial intelligence for diagnosis and treatment, they highlighted that a major disadvantage to artificial intelligence is the prioritization of accuracy over clinical evaluation and scientific validation.

### 9.3. Acceptability and Readiness for Computer Vision

The implementation of artificial intelligence in healthcare is impacted by public opinion. In a comprehensive review published by Bekbolatova et al. (2024), the results of Pew Research surveys are highlighted, emphasizing the correlation between familiarity with artificial intelligence and the expressed potential for it to benefit healthcare [259]. While readiness for artificial intelligence is growing, the need to address specific knowledge gaps within the community to increase familiarity with artificial intelligence tools is also growing. Parallel efforts are needed to develop a comprehensive understanding of legislation and guidelines for the responsible use of artificial intelligence in healthcare [259]. A recent 10-question survey focused on the use of CV in healthcare was completed by 233 providers and 50 patients and family members. The potential for the use of CV data in lawsuits (81% clinicians) and privacy breaches (50% patients) were major areas of concern [245]. Future work should focus on further exploring provider, patient, and public perceptions and knowledge needs on CV.

### 9.4. Data Needs and Considerations

Despite the impressive performance of deep learning models on general datasets, achieving accurate results in the medical domain remains challenging. This difficulty arises mainly from the substantial number of parameters in each layer of CNN models. When a sufficient amount of data is available, as found in large CV datasets like ImageNet [260] (1 million images), the model is better able to generalize and overfitting is mitigated. Acquiring a sufficient sample of labeled data for model training within the healthcare system to produce models that are generalizable and statistically fit can be prohibitively expensive. One potential solution to address overfitting is employing models with fewer parameters [261,262]. However, these compact models often struggle to capture intricate features of the dataset, resulting in reduced detection or classification accuracy. To cope with the scarcity of labeled data, data augmentation is used to generate additional training data [114,263]. While this approach partially resolves the problem, the repetition of images may lead to overfitting. Another strategy involves utilizing transfer learning, where the model is initially trained on a large dataset with available labels and then fine-tuned on the smaller medical datasets [115,264]. This approach aims to leverage pre-existing knowledge from the larger dataset to enhance the performance of the medical data. Each of these solutions is a trade-off in model performance and must be weighed in the development and testing stages. Another option to scale the development of CV models in medicine is to use available deep-learning techniques to classify, segment, and detect specific structures or abnormalities. Detectron2 [265], developed by Facebook AI Research (FAIR), offers a high-quality implementation of state-of-the-art object detection and segmentation models. MMDetection [266], another open-source PyTorch library, facilitates the utilization of pre-trained state-of-the-art models and their training on medical datasets. Torchvision, an official PyTorch library, provides general models that can be tailored for use with medical domain datasets. OpenPose [267] stands out as one of the initial open-source and real-time multi-person models designed to identify human body structures, body key points, as well as facial and hand features in visual footage. These are a sampling of available deep learning techniques, and it is important to consider their development and validation prior to use to develop subsequent CV models. Lastly, the training of CV models demands significant computing resources and expertise, including GPUs and AI specialists, which may not be readily available at every institution due to resource constrained- environments. In light of these challenges, many clinician-scientists opt to use traditional machine learning methods, like logistic regression, that limit model development. Future CV studies may explore how federated learning could expand datasets and computational resources [268].

### 9.5. Computer Vision Datasets

Object detection datasets typically consist of images with annotated bounding boxes and segmented areas depicting objects of interest. The Pascal Visual Object Classes (VOC) [269] dataset stands out as a well-known benchmark, featuring 5000 images across 20 object classes with 12,000 annotations. Another widely used benchmark, the Common Objects in Context (COCO) [270] dataset, offers a substantial dataset of 164,000 images covering 80 object classes, accompanied by 897,000 annotations, encompassing both indoor and outdoor environments. However, in the context of hospital environments, there is currently a lack of sufficient datasets capturing diverse objects under various conditions. For example, the MCIndoor2000 [223] dataset includes 2055 images of three object classes including doors, stairs, and hospital signs. The MYNursingHome [224] dataset focuses on object classification and detection in nursing homes, containing 37,500 images featuring objects commonly found in elderly home care centers, such as toilet seats, tables, and wheelchairs. The Hospital Indoor Object Detection (HIOD) dataset comprises 4417 images covering 56 object categories, including items like surgical lights, IV poles, and bedside monitors, with a total of 51,869 annotations. On average, the images in this dataset contain 10 objects spanning 6.8 object categories [225]. There are several datasets available for medical imaging purposes. The website https://www.cancerimagingarchive.net/browse-collections/, accessed on 16 February 2024, holds several publicly available datasets.

This dearth of public datasets is illustrated best by examining the large amount of literature and models developed in neonatal populations. The cumulation of this work over the past two decades has led to a point-of-care mobile application for procedural pain that has passed the feasibility stage [172]. This type of technology could greatly improve pain management not only in neonatal populations but also in adult populations. Several studies aimed at identifying chronic or outpatient pain have used the UNBC-McMaster Pain Archive [160,161,165]. While these images have aided in the development of automated models for pain detection and monitoring in adult outpatient populations, they have not facilitated the expansion of such models into the acute care setting. Public datasets of hospitalized patients across age groups to facilitate this type of modeling are needed [172].

### 9.6. Limitations of This Review

This review used a broad search strategy. That being said, a systematic review approach was not used, and it is possible that studies involving CV were not included. The CV field is rapidly expanding. Due to that expansion, this review is limited in scope and strove to highlight advances in CV for healthcare clinicians and clinician-scientists (i.e., end-users of technology).

## 10. Conclusions

This review summarizes the application of CV in healthcare, and we highlight important considerations for the use of CV in healthcare including privacy, safety, and ethical factors. The overall goal is to improve the patient and clinician journey within the industry. There continues to be a paucity of data to train CV and for it to catch up to other industries in its application; substantial work is needed to overcome to barriers of privacy and safety considerations.

## Figures and Tables

**Table 1:** Display of different types of machine learning models used in computer vision.

Approach	Supervision	Machine Learning Model	Description
Deep Learning	Supervised	Convolutional Neural Network	Mostly used for classification and segmentation. It includes wide range of model architectures such as ResNet, VGG-net, and AlexNet.
		Mask-region-based Convolutional Neural Network	CNN type primarily employed for detecting objects in input images.
		YOLO	CNN types primarily employed for image segmentation or classification.
		U-net	Type of CNN mainly used for image segmentation.
		Gated recurrent unit	Type of recurrent neural network tailored for modeling time dependent data to address long-range dependencies in sequential data.
		Long short-term memory (LSTM)	
		Vision transformer	Novel category of CNNs. Adopts transformer architecture commonly used in NLP and shows high performance in image classification benchmarks.
	Unsupervised	Convolutional Deep Belief Network (CDBN)	Type of deep generative models that is constructed by stacking max-pooling Convolutional Restricted Boltzmann Machines (CRBMs).
		Autoencoder	A type of neural network that specializes in learning to convert data into a compact and efficient representation, often employed for the purpose of dimensionality reduction.
Traditional	Supervised	*k*-nearest neighbors	Assigns class labels or values according to the distance of the input data to the *k*-nearest neighbors in the training data.
		Binary Tree	Decision-making algorithm that navigates the tree from root to leaf to make decisions based on specific features or attributes.
		Naïve Bayes	Probabilistic machine learning algorithm that classifies data based on the conditional independence between every pair of features.
		Support vector machine (SVM)	It uses the kernel trick to find a linear decision boundary to separate input data in the transformed space.
		Fuzzy Inference System	A computational model that uses fuzzy logic to perform reasoning on uncertain or imprecise information.
		Fisher’s linear discriminant analysis	Classifies input data based on linear combination of features that represent items in each class.
		Linear Mixed Model	An extension of simple linear models that allow fixed and random effects, useful for complex data
		Logistic/linear regression	This is a statistical model that uses the logistic function to predict the probability of a specific class.
	Supervised and Unsupervised	Random forest	Ensemble learning method that comprises multiple trees trained on random subsets of data. The final prediction is aggregated from all trees.
		Neural network	It is a conventional machine learning model employed for classification and regression. In comparison to existing deep methods, it exhibits lower accuracy.
		Singular Value Decomposition (SVD)	It decomposes the input feature space into 3 generic and familiar matrices.
	Unsupervised	Fuzzy C-means	A computational model that uses fuzzy logic to perform reasoning on uncertain or imprecise information
		Gaussian Mixture Model Segmentation	It uses Gaussian distribution to partition pixels into similar segments

Table 1 outlines the types of machine learning models used in CV algorithm development. As computer vision has progressed over the years, the use of deep supervised models has increased. This innovation includes the use of the transformers and autoencoders listed above.

**Table 2:** Uses and categorizations (themes) of computer vision in industry outside healthcare.

Industry	Themes			
	Job Enhancement	Surveillance (S) Monitoring (M)	Automation	Augmented Reality
Agriculture		Monitor crops (M) [15,38,39]	Weed detection and elimination https://weedbot.eu/, acessed on 1 July 2023	
Animal control		Wildlife monitoring (M) [49] Farm animals monitoring (M) [50]		
Art security		Forgery detection (S) [35,36,37,51]		
Automotive		Parking lot analysis (M) [52]	Self-driving cars https://tesla.com/, accessed on 15 July 2023	
Digital design	Video enhancement [53] Image/video deblurring [54]			
Education		Cheating prevention (S) [55]		
Engineering	Importing real life objects into modeling software [56]			
Food service		Reduce food waste in restaurants (M) [57]	Robotic food delivery https://starship.xyz/, accessed on 15 July 2023	
Gaming				Xbox Kinect [58] Gesture based gaming [59]
Government	Control traffic lights [60]	Detecting natural disasters (M) [61]		
Insurance	Insurance appraisals [10,62,63]			
Law enforcement	Forensic analysis[64]	Facial recognition in large crowds (S) [65] Identity verification (S) [66] Detect dangerous situations (S) [67]	Speeding enforcement [14]	
Manufacturing			Defective products on an assembly line [68]	
Manufacturing	Workplace inspection [69]			
Medical	See Table 3			
Military		Terrain Reconnaissance (S) [70]	Automate military drones [71]	
Movie	Film movie restoration [72]			
Retail	Customer behavior analysis (Traffic volume heatmaps) https://n-ix.com/, accessed on 1 July 2023 Staff demand for optimal shift assignments https://n-ix.com/, accessed on 1 July 2023	Detecting defective products (M) [73]	Floor-cleaning robots [74] Detecting restock [75] Identifying retail products at sale https://n-ix.com/, accessed on 1 July 2023	Trying on clothes virtually [43,44,45] Virtual testing and visualization of products in their intended space [76]
Robotics			Helping robots move around environment https://inbolt.com/, accessed on 1 July 2023	
Social media	Social media recommendation [77]		Inappropriate content detection [78]	
Space		Tracking asteroids and debris (M) [79]	Landing spacecraft [80,81]	
Sports	Finding game highlights in videos [82] Sport performance analysis [33,34]		Ball tracking in sports [83,84] Refereeing automation [33]	
Tech		Facial recognition personal mobile devices (M) [85]		Language translation of video and images [86] Azure Kinect [87] Apple vision https://apple.com/apple-vision-pro/, accessed on 15 July 2023

Table 2 outlines the use of computer vision across different industries (first column) and how that use is categorized into identified themes. This is not an exhaustive list. This table was created to illustrate how other industries use computer vision to improve efficiencies across systems and could provide insight into how computer vision could be used in healthcare.

**Table 3:** CV applications in healthcare.

Areas	Citations	Image Type	Deep Model	Application
Medical imaging and diagnosis	[98,99,100,101,102,103]	CT, F-FDG PET/CT, Chest X-rays	Mask-RCNN, CNN, Transformer, SVM, random forest, *k*-nearest neighbor	Lung cancer, tuberculosis
	[104,105,106]	Iris, cellular retinal, fundus	Binarytree, Random Forest, SVM, neural network, CNN	Changes in vision related to diabetes
	[107,108,109]	HD microscope	Vision transformer, CNN	Cervical cancer
	[97,110,111,112,113,114,115,116,117,118,119]	Mammogram, whole slide images, hematoxylin, eosin	YOLO, CNN, random forest, SVM, decision tree, Naïve Bayes, Logistic linear classifier, Linear discriminant classifier, Fischer’s Linear Discriminant analysis, *k*-nearest neighbor, Autoencoders	Breast cancer, data augmentation
	[120,121,122,123]	Dermoscopic image	CNN, Gated recurrent unit	Skin cancer detection/segmentation
	[124,125,126,127]	Endoscopic images, hematoxylin & eosin, whole-slide images	CNN, transformer, U-net	Colorectal, gastrointestinal cancer
	[128,129,130,131,132,133]	Chest X-rays	CNN, transformer, logistic regression	COVID-19 diagnosis Age estimation in unidentified patients
	[134]	Whole slide images	Vision transformer	Subtyping of papillary renal cell carcinoma
	[25,29,30,98,127,135,136,137,138,139,140,141,142,143,144,145,146,147,148,149,150,151]	MRI, Histogram, CT, X-ray, ultrasound, PET	CNN, Naïve Bayes, Random Forest, Neural Networks, SVM, *k*-nearest neighbor, Decision Tree, logistic function, Naive Bayes, Fuzzy *k*-means	Cancers (brain, bladder, breast, liver, lung, pancreas, prostate, other), CT reconstruction, Alzheimer’s Disese, intracranial hemorrhage
	[152,153,154,155]	Dual energy X-Ray absorptiometry (DEXA) X-ray	SVM YOLOv8.0, Detectron2, several others (see systematic review)	Lumbar spine fractures Pediatric fractures Overall fracture identification
Delirium	[156]	Surveillance images	CNN, *k*-nearest neighbors	Delirium monitoring
Pain, Agitation, Stress, Level of sedation	[157,158,159,160,161,162,163,164,165,166,167,168,169,170,171,172,173,174,175,176,177,178,179,180]	Surveillance images, depth image, face images, pain datasets	YOLO, Mask-RCNN, CNN, CDBN, SVM, LSTM, LMM, Neural Network	Activity recognition, detection of pain and discomfort, stress, automated facial analysis for grimacing, agitation, eye localization, depression, anxiety, stress levels, AU
Patient deterioration	[181,182]	Color videos	Logistic/linear Regression	Deterioration prediction using AUs
Mechanical ventilation	[183,184,185]	Chest X-rays, ICU videos	U-net, YOLO, TL, Feature descriptor	Need for mechanical ventilation, detect and recognize ventilation objects and positioning, estimate lung volume
Mobility	[186,187]	ICU video images	CNN, YOLOv2	Patient mobilization activities in ICU, NIMS
Patient safety	[188,189,190,191,192,193,194,195]	Surgical videos, depth images, video recordings	OpenPose, Yolo, CNN, Mask-RCNN	Surgical team behavioral analysis, patient mobilization activities, hand hygiene, ICU staff monitoring, assessing situational awareness
Surgical assistance	[194,196,197,198,199]	Surgical activity images, OR videos	CNN	Robot-assisted surgery, situational awareness in OR
Neurological, neurodevelopmental, psychiatric disorders	[142,162,174,178,200,201,202,203,204,205,206,207,208,209],	Whole-body video recording, MRI, PET, patient images	Detectron2, OpenPose, CNN, *k*-nearest neighbor, SVM, K-SVD, Bayesian Networks	Analysis of gain synchrony, balance, Infant neuromotor risk, neurodegenerative disease, behavioral analysis in ASD and ADHD, facial expression in depression, facial weakness
Remote monitoring, telemedicine	[210,211]	Surveillance images	Deep reinforcement learning, CNN	In-home elbow rehabilitation
Data security and privacy	[114,212,213,214]	X-rays, MRI	CNN, Fuzzy CNN	Privacy protections for deep learning algorithms containing medical data
Fall detection	[215,216,217,218,219]	Surveillance images	Gaussian Mixture Model, CNN Segmentation, AlphaPose, OpenPose, LSTM	Human fall detection
Hospital scene recognition	[192,220,221,222,223,224,225,226]	Indoor images of ICU, hospital, nursing home; pediatric ICU videos	YOLO, CNN, SVM, CATS	ICU and hospital indoor object detection, hand hygiene, ICU activity measurement

Table 3 describes the varying uses of CV technology in healthcare and outlines the image captures, machine learning models used, and the focus area. This is not an exhaustive list. Abbreviations: AU: action unit; ASD: autism spectrum disorder; CATS: Clinical Activity Tracking System; CNN: Convolutional Neural Network; NIMS: Non-Invasive Mobility Sensor; OR: operating room.

## Data Availability

Search results and datasheets of extracted data are available upon request to the corresponding author.
